# Challenging in pulmonary thromboembolism diagnosis in patients with disproportionate pulmonary hypertension and severe mitral stenosis: Report of two cases

**DOI:** 10.1002/ccr3.8597

**Published:** 2024-03-07

**Authors:** Naser Khezerlouy‐Aghdam, Mehrnoush Toufan Tabrizi, Kia Seyed Toutounchi, Amirreza Jabbaripour Sarmadian, Shahab Masoumi

**Affiliations:** ^1^ Cardiovascular Research Center Tabriz University of Medical Sciences Tabriz Iran; ^2^ Faculty of Medicine Tabriz University of Medical Sciences Tabriz Iran

**Keywords:** echocardiography, mitral stenosis, pulmonary hypertension, thromboembolism

## Abstract

**Key Clinical Message:**

Before valvular interventions, echocardiography, especially the TEE or the ventilation/perfusion scan, should be performed to detect silent PTE and set a more accurate treatment and surgical plan.

**Abstract:**

Pulmonary hypertension (PH) is a progressive and critical disease that can be caused by mitral stenosis (MS). Some of these patients present with disproportionate PH, which is an uncommon phenomenon and is considered a challenging diagnostic and treatment process. In these patients, other causes may also play a role in developing PH. This report presented two cases with disproportionate PH and severe MS who were scheduled for percutaneous mitral valvuloplasty (PMV). The pre‐procedural echocardiography revealed systolic pulmonary artery pressure (sPAP) of 90 and 120 mmHg, mitral valve area of 0.80 and 0.55 cm^2^ by three‐dimensional (3D) planimetry, and diastolic pressure gradient (DPG) of 13 and 18.8 mmHg, respectively. Furthermore, in the first patient, 3D transesophageal echocardiography (TEE) revealed multiple saddle‐type organized thrombi in the proximal parts of the right and left pulmonary arteries, extending to the distal branches. In the second patient, 3D TEE revealed a large, relatively fresh, flow‐limiting thrombosis in the proximal part of the right pulmonary artery. The diagnosis of pulmonary thromboembolism (PTE) in both patients was confirmed by CT angiography. In both patients, the valves were surgically repaired, while all thrombi were removed from the cardiac chambers and pulmonary vessels during surgery. In addition, patients underwent warfarin therapy orally. They were followed up 6 months after the intervention, and their clinical symptoms had improved significantly.

## 1 INTRODUCTION

Pulmonary hypertension (PH) is a progressive and critical disease characterized by an increase in systolic pulmonary artery pressure (sPAP) and mean pulmonary arterial pressure (mPAP), ultimately leading to right heart failure and death, with an estimated prevalence of 1% worldwide, increasing up to 10% in elderly patients (over 65 years).^1^, ^2^ It is generally defined as an mPAP >20 mmHg (Previously 25 mmHg), measured invasively through right heart catheterization. In addition, at different stages of the disease, an increase in pulmonary vascular resistance (PVR), pulmonary artery wedge pressure (PAWP), and even diastolic pressure gradient (DPG) can be observed.^3^, ^4^, ^5^

Patients with severe mitral stenosis (MS), defined as a mitral valve area of less than 1.5 cm^2^, are highly susceptible to PH. PH patients with MS suffer from the post‐capillary type of PH and are placed in Group 2 of the PH classification due to their left heart disease.^5^, ^6^, ^7^ Some of these patients present with severe PH, defined as sPAP exceeding 70 mmHg or the mPAP exceeding 40 mmHg, unresponsive to standard medical therapy and valvular interventions, which is an uncommon phenomenon and is considered a challenging diagnostic and therapeutic process. Pre‐capillary causes may also play a role in developing PH, so they should be carefully considered in these patients.^3^, ^4^, ^5^ This report presents two challenging cases of disproportionate PH and severe MS, of which pulmonary thromboembolism (PTE) was detected in pre‐procedural investigations as a contributing factor in developing PH.

## 2 CASE HISTORY/EXAMINATION

The first patient was a 62‐year‐old woman referred with severe MS who was scheduled for percutaneous mitral valvuloplasty (PMV). On clinical evaluations, she was suffering from dyspnea, palpitation, and chest pain, and she was in stable hemodynamic condition with tachycardia and a normal state of consciousness. Her blood pressure was 125/75 mmHg; her heart rate was 105 bpm; her body temperature was 37.0°C; her respiratory rate was 19; and her oxygen saturation was 93% without oxygen supplementation. On physical examinations, she had jugular vein distention, loud secondary heart sound, diastolic murmur in the mitral region, opening click alongside S_1_ and S_2_, and bilateral lower extremity edema.

## 3 METHODS (DIFFERENTIAL DIAGNOSIS, INVESTIGATIONS, AND TREATMENT)

Atrial fibrillation (AF) rhythm was observed in the electrocardiography (ECG) obtained from the patient with a heart rate of 85 per minute. For further evaluations of cardiac chambers and valves, as patient was the known case of MS, transthoracic echocardiography (TTE) was performed and revealed severe PH, with an sPAP of 90 mm Hg. Echocardiographic parameters related to cardiac chambers and valves are shown in Table 1. Multiple thrombi were observed in the left atrium, right atrium, and right auricle.

**TABLE 1:** Echocardiographic Parameters of Both Patients.

Parameters	Findings in patient 1	Findings in patient 2
Systolic pulmonary artery pressure (sPAP)	90 mmHg	120 mmHg
Diastolic pressure gradient (DPG)	13 mmHg	18.8 mmHg
Left atrium		
Size	Enlarged	Enlarged
Left atrial volume index (LAVI)	42 cc/m^2^	102 cc/m^2^
Left ventricular		
Size	Normal size with mild left ventricular hypertrophy (LVH)	Normal size with mild left ventricular hypertrophy (LVH)
Left ventricular ejection fraction (LVEF)	50%	50%
Right atrium		
Size	Normal size	Enlarged
Right atrium area (RAA)	14.5 cm^2^	26.6 cm^2^
Right ventricle		
Size	Normal size	Enlarged
Right ventricular diastolic dimension (RVDD)	38.6 mm	59 mm
Tricuspid annular plane systolic excursion (TAPSE)	14.8 mm	15 mm
Mitral valve		
Mitral valve area	0.31 cm^2^ by 3D‐TEE, 0.80 cm^2^ by 3D‐planimetry, and 0.52 cm^2^ by PISA	0.63 cm^2^ by 3D‐TEE, 0.69 cm^2^ by 3D‐planimetry, and 0.55 cm^2^ by PISA
Mitral stenosis (MS)	Severe MS	Severe MS
Mitral regurgitation (MR)	Moderate MR	Severe MR
Tricuspid valve		
Tricuspid regurgitation (TR)	Severe TR	Severe TR
Tricuspid regurgitation gradient (TRG)	75 mmHg	100 mmHg

**FIGURE 1:**
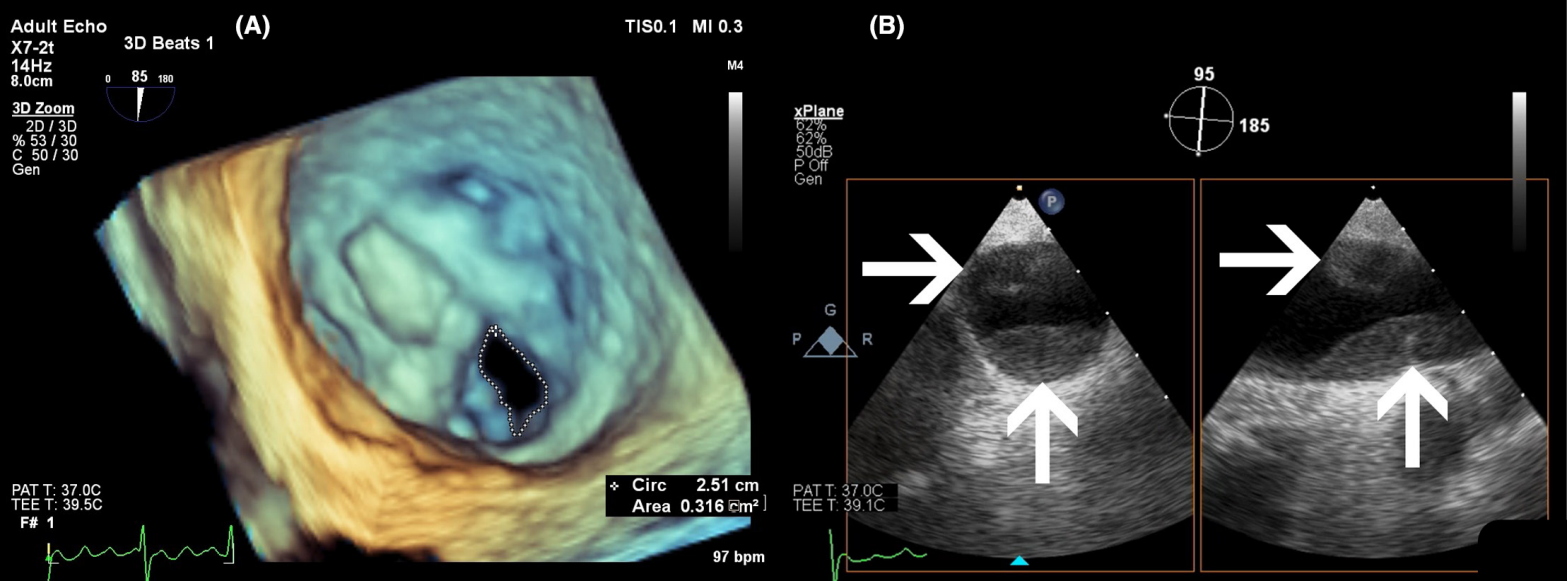
(A) 3D‐TEE mitral valve view demonstrating severe MS with a mitral valve area of 0.31 cm^2^. (B) X‐Plane TEE view of the pulmonary artery revealing large thromboses as shown by white arrows in the proximal part of the right pulmonary artery.

The mitral valve area was evaluated as 0.31 cm^2^ by three‐dimensional (3D)‐transesophageal echocardiography (TEE), 0.80 cm^2^ by 3D‐planimetry, and 0.52 cm^2^ by PISA confirming severe MS (Figure 1, Video S1). Furthermore, both the right and left pulmonary arteries were dilated, with diameters of 26.4 and 28.9 mm, respectively. Trace pericardial effusion and thick fat pads were also detected surrounding the heart.

**FIGURE 2:**
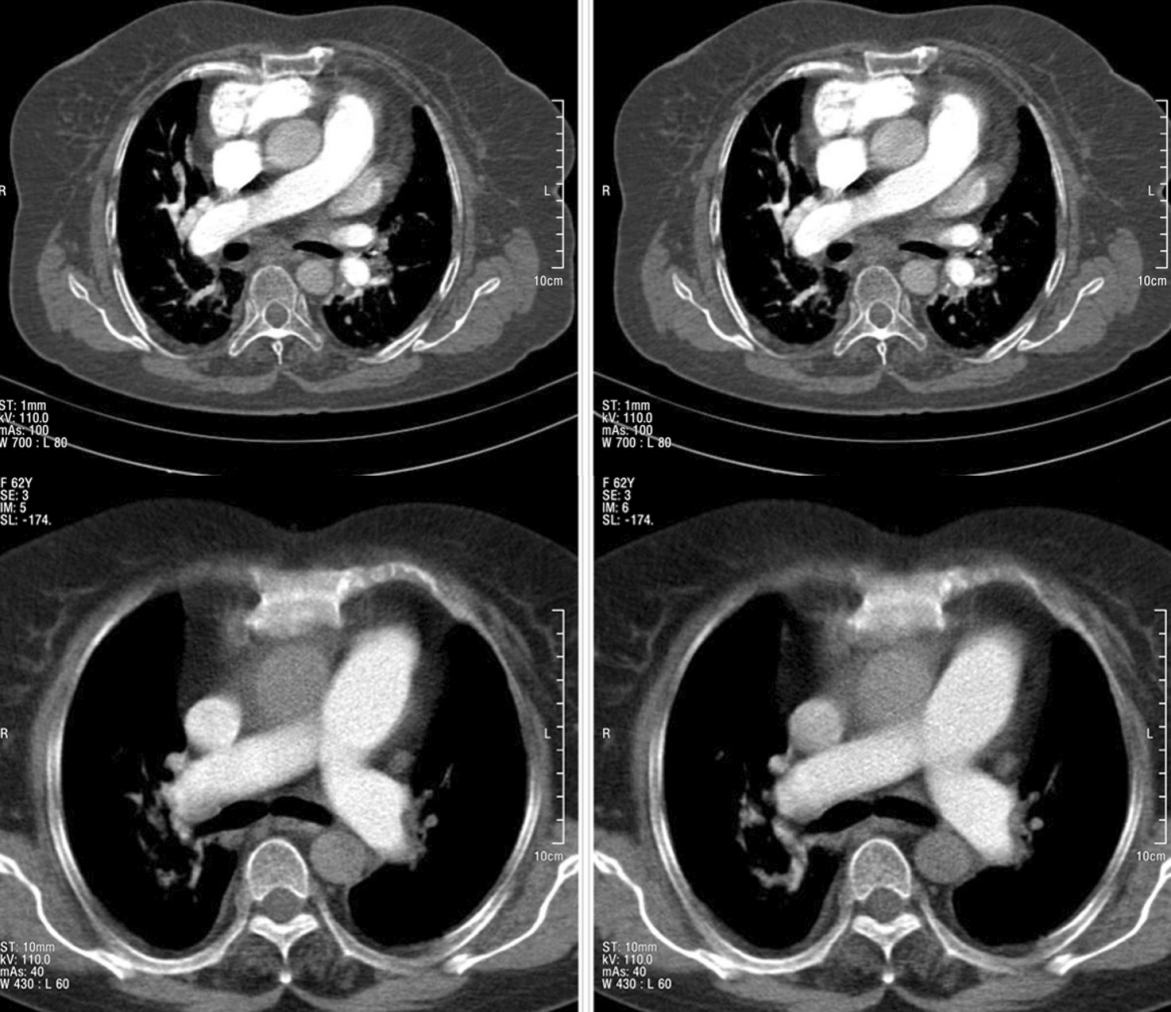
CT angiography revealing right and left PTE.

3D‐TEE revealed multiple saddle‐type organized thrombi in the proximal parts of the right and left pulmonary arteries, extending to the distal branches, leading to a diagnosis of PTE (Figure 1). Following echocardiographic findings, computed tomography (CT) angiography was performed for further evaluation, which confirmed PTE (Figure 2).

The patient underwent surgical repair of the mitral valve, and all thrombi were also removed from the proximal right and left pulmonary arteries by thrombectomy during the surgery. Because the patient had many distal branch thrombosis, while we removed the ones in proximal sections, we had to leave the distal branches for anticoagulant therapy after surgery. Therefore, warfarin was administered at 2.5 mg daily, keeping the international normalized ratio (INR) at 3–3.5. Post‐surgery ECG revealed normal sinus rhythm, and TTE revealed a decrease in sPAP from 90 to 40 mmHg and a decrease in the DPG from 13 to 4 mmHg.

## 4 CASE HISTORY/EXAMINATION

The second patient was a 53‐year‐old man referred with severe MS who was scheduled for PMV. On clinical evaluations, he was suffering from paroxysmal nocturnal dyspnea, fatigue, and palpitation, and he was in stable hemodynamic condition with tachycardia, tachypnea, and a normal state of consciousness. His blood pressure was 135/75 mmHg; his heart rate was 103 bpm; his body temperature was 36.8°C; his respiratory rate was 22; and his oxygen saturation was 92% without oxygen supplementation. On physical examinations, he had jugular vein distention, loud secondary heart sound, diastolic murmur in the mitral region, opening click alongside S_1_ and S_2_, S_3_ gallop, and bilateral lower extremity edema.

## 5 METHODS (DIFFERENTIAL DIAGNOSIS, INVESTIGATIONS, AND TREATMENT)

**FIGURE 3:**
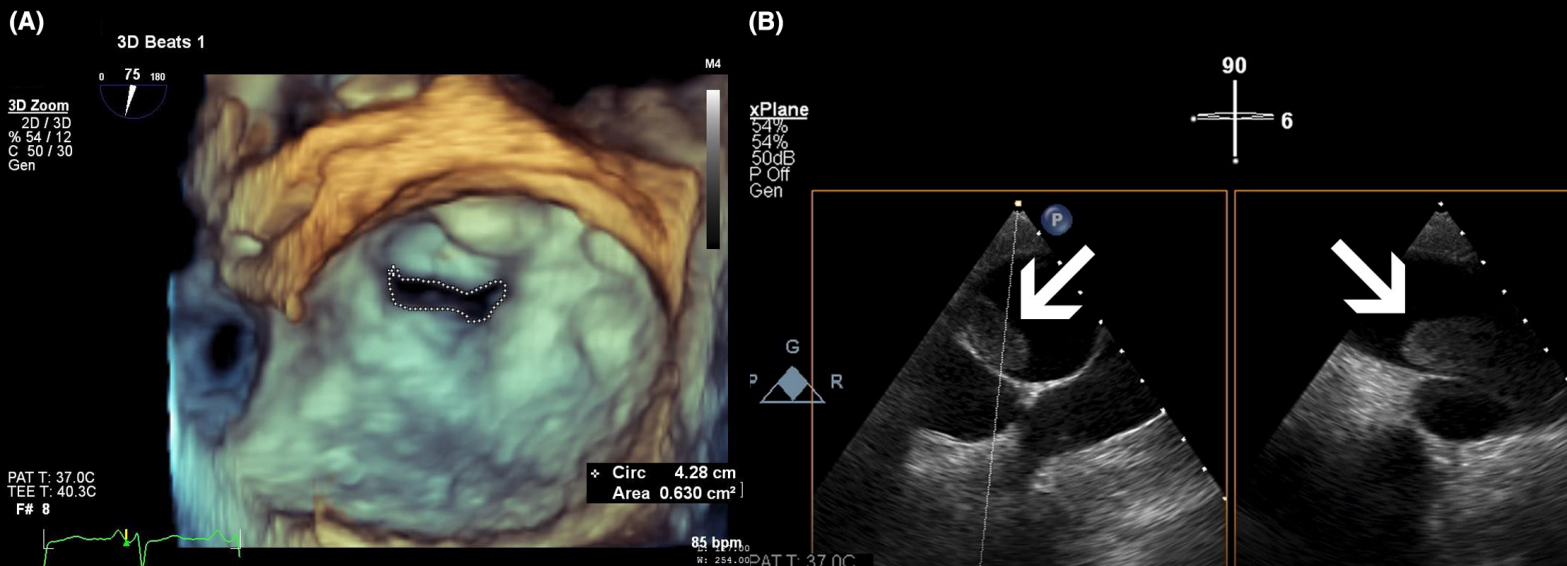
(A) 3D‐TEE mitral valve view demonstrating severe MS with a mitral valve area of 0.63 cm^2^. (B) X‐Plane TEE view of the pulmonary artery revealing large thromboses as shown by white arrows in the proximal part of the right pulmonary artery.

Normal sinus rhythm was observed in the ECG obtained from the patient with a heart rate of 91 per minute. For further evaluations of cardiac chambers and valves, as the patient was the known case of MS, TTE was performed and revealed severe PH, with an sPAP of 120 mmHg. Echocardiographic parameters related to cardiac chambers and valves are shown in Table 1. There was no evidence of thrombosis in the atriums and ventricles. The mitral valve area was evaluated as 0.63 cm^2^ by 3D‐TEE, 0.69 cm^2^ by 3D‐planimetry, and 0.55 cm^2^ by PISA confirming severe MS (Figure 3, Video S2). Furthermore, the right pulmonary artery was dilated, with a diameter of 38 mm. Right side pleural effusion, trace pericardial effusion, and ascites were also detected.

**FIGURE 4:**
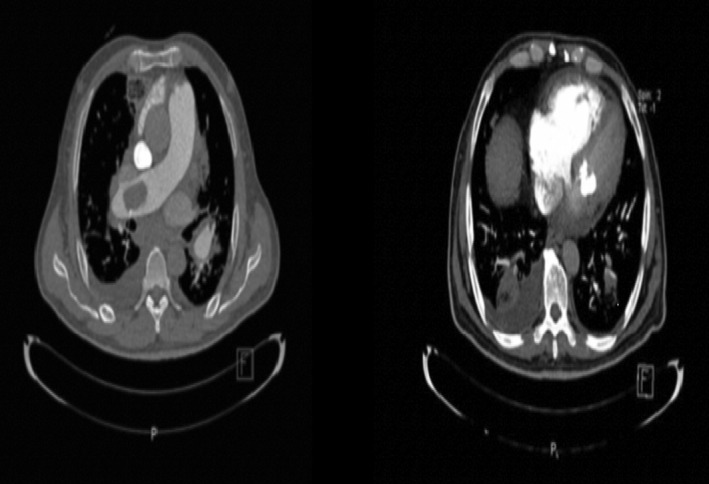
CT angiography revealing right PTE.

3D‐TEE revealed a large, relatively fresh, flow‐limiting thrombosis in the proximal part of the right pulmonary artery (Figure 3). Therefore, CT angiography was performed for further evaluation, which confirmed PTE (Figure 4).

Considering the high Wilkins score (more than 12), surgical mitral valve replacement was performed and the thrombosis was removed from right pulmonary artery by thrombectomy during the surgery. Post‐surgery TTE revealed a decrease in sPAP from 120 to 35 mmHg and a decrease in the DPG from 18.8 to 5 mmHg. After surgery, furosemide 40 mg every 12 h and warfarin 2.5 mg daily was administered, keeping the INR at 3–3.5. Following stable vital signs 10 days postoperatively, the patient was discharged on lifelong warfarin.

## 6 FOLLOW‐UP AND OUTCOME

Both patients were followed up 6 months after the intervention, and their clinical symptoms had improved significantly. The first patient only complained of mild exertional dyspnea, and she no longer had any signs and symptoms in‐favor‐of right heart failure. In addition, CT angiography revealed that all the small distal thrombi in the pulmonary arteries were resolved. The second patient no longer had any signs and symptoms in‐favor‐of right heart failure. In addition, CT angiography revealed that the thrombosis in the right pulmonary artery was resolved.

## 7 DISCUSSION

According to the Sixth World Symposium on pulmonary hypertension and world health organization, PH is classified into five major categories, considering pathophysiological mechanisms, clinical presentations, and therapeutic management, including Group 1: pulmonary arterial hypertension, Group 2: PH due to left heart disease (PH‐LHD), Group 3: PH due to lung disease, Group 4: chronic thromboembolic pulmonary hypertension (CTEPH), and Group 5: PH with unclear and or multi‐factorial mechanisms. Furthermore, it can also be classified into three groups in terms of hemodynamic characteristics^1^, ^3^, ^4^:

1. Pre‐capillary PH is due to primary hypertension in the pulmonary arterial system and is observed in Groups 1, 3, 4, and 5. It is defined as mPAP >20 mmHg, PAWP ≤15 mmHg, and PVR ≥3 wood units (WU).
2. Isolated post‐capillary PH (IpcPH) is due to hypertension in the pulmonary venous and pulmonary capillary systems and is observed in Groups 2 and 5. It is defined as mPAP >20 mmHg, PAWP >15 mmHg, and PVR <3 WU.
3. Combined pre‐ and post‐capillary PH (CpcPH) is more complicated than pre‐capillary PH and IpcPH and should be considered if DGP ≥7 mmHg. It is defined as mPAP >20 mmHg, PAWP >15 mmHg, and PVR ≥3 WU.

This report presented two cases with severe CpcPH, in which sPAP was 90 and 120 mmHg and DPG was evaluated as 13 and 18.8, respectively. As mentioned, the post‐capillary cause of PH in our patients was severe MS, in which the mitral valve area was assessed 0.80 and 0.63 cm^2^ by 3D planimetry, respectively. As discussed above, PH has various etiologies and the most common one is left heart disease (PH‐LHD), accounting for approximately 50%–85% of all PH cases, one of the most common causes of which is MS.^3^, ^5^ Increased left heart pressure, secondary to severe MS, leads to pulmonary venous hypertension, gradual changes in the pulmonary vascular bed, and remodeling, eventually, the development of PH and progression to its more severe forms with poor prognosis. In the early stages of post‐capillary PH following severe MS, PH is passive in nature and develops secondary to increased outflow pressure due to valvular disease, which mostly resolves if the underlying cause and valvular disease are treated. If it is not treated and remains chronic, a pre‐capillary component may be added and progress to CpcPH, which has an active progressive nature, leading to vascular remodeling and significantly increasing the morbidity and mortality.^5^, ^6^, ^8^

In both patients, pre‐capillary PH was caused by thromboembolism in the pulmonary arteries, so they are placed in Group 4 of the PH classification (CTEPH), which is considered a critical group of PH, associated with high morbidity and mortality.^9^, ^10^ The overall incidence and prevalence of CTEPH were estimated to be 0.9–39 and 14.5–144 per 1 million adults, respectively.^11^ AF rhythm may lead to thromboembolic events not only in both atria but also throughout the circulatory system, which is commonly observed among patients with MS.^12^ It was observed in the first patient, which was resolved after the intervention and turned into a normal sinus rhythm, so in this patient the cause of thromboembolism was probably the AF rhythm. However, the second patient had normal sinus rhythm since admission.

CTEPH mainly develops as a late complication of PTE leading to vascular remodeling. In a systematic review and meta‐analysis study by Zhang et al.,^10^ the overall incidence of CTEPH following acute PTE, with a median follow‐up from 6 to 94.3 months, was evaluated as 3.13%. However, considering that PTE can be clinically silent in a significant number of patients, its timely diagnosis is challenging and the exact incidence and prevalence may be higher than the estimated values.^10^, ^11^

As mentioned, PTE was incidentally detected on TEE in both patients, so pre‐procedural TEE especially 3D type should be performed in patients who are candidates for valvular interventions, as it shows thrombus in pulmonary arteries and confirm the diagnosis of PTE.^13^ However, ventilation/perfusion (V/Q) scan is the screening test of choice, as a normal scan rules out CTEPH, and an abnormal scan will require further investigations such as CT angiography.^9^

## 8 CONCLUSION

CpcPH with pre‐capillary PH of CTEPH and post‐capillary PH of MS is a rare phenomenon; however, it can be present in patients with disproportionate PH and MS who do not respond to interventions as PTE can be silent in these patients. Therefore, it is suggested to perform further investigations for all patients before valvular interventions such as TEE and V/Q scans in order to detect silent PTE and set a more accurate treatment and surgical plan.

## AUTHOR CONTRIBUTIONS

**Naser Khezerlouy‐Aghdam:** Conceptualization; methodology; project administration; supervision. **Mehrnoush Toufan Tabrizi:** Conceptualization; methodology; supervision; validation. **Kia Seyed Toutounchi:** Data curation; investigation; writing – original draft. **Amirreza Jabbaripour Sarmadian:** Writing – original draft; writing – review and editing. **Shahab Masoumi:** Data curation; validation; writing – original draft; writing – review and editing.

## FUNDING INFORMATION

There was no financial support or funding for this research.

## CONFLICT OF INTEREST STATEMENT

The authors declare no financial and non‐financial competing interests related to this work.

## ETHICS STATEMENT

This study was performed according to the principles outlined by the World Medical Association's Declaration of Helsinki on experimentation involving human subjects, as revised in 2000, and was approved by the Tabriz University of Medical Sciences ethics committee with the approval number IR.TBZMED.REC.1402.385 on 2023/08/20.

## CONSENT

Written informed consent was obtained from the patient to publish this report in accordance with the journal's patient consent policy.

## Supporting information


Video S1

Video S2

## Data Availability

Data are available from the corresponding author on reasonable request.
